# Prevalence and belief in the continuation of female genital cutting among high school girls: a cross - sectional study in Hadiya zone, Southern Ethiopia

**DOI:** 10.1186/1471-2458-13-1120

**Published:** 2013-12-05

**Authors:** Mulugeta Tamire, Mitike Molla

**Affiliations:** 1 School of Public Health, Addis Ababa University, P.O.Box.9086, Addis Ababa, Ethiopia

## Abstract

**Background:**

Female Genital Cutting is a cultural practice among many ethnic groups in Ethiopia that has affected many girls over the past centuries. Although the trend is slowly decreasing in Ethiopia, the magnitude is still very high as the procedure has no known benefit but has many consequences. The objective of this study was to assess the prevalence and belief in the continuation of FGC among High School Girls in Hadiya Zone.

**Methods:**

A cross-sectional quantitative survey was carried out among high school girls in Hadiya Zone from January to February 2011. A multi-staged cluster sampling method was used for sample selection. In total, 780 girls completed a self-administered questionnaire for this study. Statistical analysis was done using bivariate and multivariate logistic regression.

**Results:**

Of 780 high school girls, 82.2% were circumcised at a mean age of 11(±2.3) years. Half of the total participants responded that FGC was being practiced in their village. About 60% of the circumcisions were performed by traditional circumcisers while health professionals had performed 30% of them. A few of the circumcised girls (9.4%) supported their status as a circumcised girl, but only 5% believe in the continuation of FGC. The odds of being cut was higher among girls whose fathers and mothers had educational status under high school level (AOR = 2.04; 95% CI: 1.25, 3.09) and (AOR = 1.84; 95% CI: 1.01, 3.38) respectively when compared to those whose parents had attended high school and above. The odds of believing in the continuation of FGC was 2.33(95% CI: 1.01, 5.33) times higher among those who responded that FGC was practiced in their areas.

**Conclusion:**

While there is an urgent need to stop the practice of FGC in Hadiya Zone, cultural beliefs related to the hygiene of female genitalia and other social factors contribute to sustaining the practice. Local organizations in collaboration with religious institutions and community leaders should work together to engage in a process of change within the entire community by arranging awareness creation programmes on the harmfulness of the practice especially in the rural areas of the zone.

## Background

Female Genital Mutilation (FGM), also known as Female Genital Cutting (FGC), consists of all procedures that involve partial or total removal of the external female genitalia or other injury to the female genital organs whether for cultural or other non-medical reasons [1-3]. An estimated 100 million to 140 million girls and women worldwide have undergone female genital mutilation/cutting (FGM/C) and more than three million girls are at risk for cutting each year on the African continent alone [4]. Although the practice is highly prevalent in western, eastern, and north-eastern Africa, it also occurs in some countries of Asia and Middle East and certain immigrant communities in North America, Europe and Australia [5,6]. In Africa, there was a significant regional and geographical variation of prevalence of the practice ranging from nearly 90% or higher in Egypt, Eritrea, Mali and Sudan to less than 50% in the Central African Republic and Côte d’Ivoire, to 5% in the Democratic republic of Congo and Uganda [6-8].

While there is no religious base for this practice, most people think it is performed for religious reasons. FGM dates back over 2,000 years and exists nowadays across most religious, racial and social boundaries, supported by centuries of tradition, culture and false beliefs [9]. As FGM is a cultural practice, efforts to end it require understanding and changing the beliefs and perceptions that have sustained the practice over the centuries [4].

Despite some 25 years of efforts to ban FGM in many countries around the world, the practice is still far from being eradicated [10,11]. In Ethiopia, the prevalence of FGM was 80% in 2000 and only a 5% decrease had been observed in 2005. During these years, the occurrence in Ethiopia’s Southern Nations, Nationalities and Peoples Region (SNNPR), of which the Hadiya Zone is a part, was 71% and 74.8%, respectively [12,13]. According to a follow-up survey in 2006, the rate of decrease among Hadiya was only 5.2%, and the prevalence was 70.9% [14], indicating the need for further studies and interventions to address the problem.

Where previous studies have focused on mothers' perspectives only, the aim of this study is to assess the prevalence and the beliefs of female students regarding the continuation of FGM. So, obtaining the data from the girls, themselves victims, who will be the future mothers, will expand on the literature and give direction to policy makers and other interested persons. The findings of this study will also help to design appropriate interventions to halt the practice among the new generation of females in the area.

## Methods

### Study area and population

The study was conducted in Ethiopia’s Hadiya Zone, which is situated at the margin of the Great Rift Valley in the north-western part of SNNPR. The zone is 230 km south-west of Ethiopia’s capital city, Addis Ababa. According to the 2007 population and housing census, the zone has an estimated population of 1,243,776, 50% of whom are female. The zone is divided into 10 rural woredas (the lowest administrative level) and one city administration as administrative political units. At the time of writing this study, there are 12 public high schools (grade 9 and 10) in the zone, with a total number of 25,822 students between them, 41% of whom are female.

### Study design and period

A cross-sectional quantitative study was conducted to determine the prevalence and belief in the continuation of FGM among high school girls in the Hadiya Zone from January to February, 2011.

### Sample size and sampling procedures

A sample of 797 high school girls was calculated using the formula for a single population proportion for cross-sectional study with a 95% confidence level, 5% of margin of error and a FGC prevalence of 62% among women of age 15–19 years in Ethiopia from the Ethiopian Demographic and Health Survey (EDHS)-2005 [13] and considering design effect of two as it was a multi-staged sampling procedure and non-response rate of 10%.

A multi-stage cluster sampling technique was used for selecting the girls to be included in the survey. Among the 12 governmental high schools found in the zone, four schools were selected by random sampling; the sample’s 797 female students were allocated proportionately to the size of the four schools. Then, participants were selected using systematic sampling technique from each school based on their class attendance rosters.

### Data collection techniques

A structured, self-administered questionnaire, which contained socio-demographic characteristics like age, ethnicity and religion, as well as parental educational status and occupation, knowledge, and beliefs, was used to collect data. Pre-tests were conducted in Heto High School, which is not included in the study, using 5% of the questionnaires prepared in English, translated to Ethiopia’s national language Amharic and back to English 15 days before data collection. Subsequently, some corrections were made to the questionnaire. Two teachers from each selected school were recruited and trained for one day on data collection techniques to give explanations, collect data and check completeness and report to the principal investigators. The questionnaires were distributed to the selected students in the classroom and collected on the same day to avoid information contamination.

### Data processing and analysis

After data collection was completed, data entry and cleaning were done using EPI- Info version 3.5.1 and exported to SPSS 16.0 package for analysis. The first step before analysis was to visualize the general feature of the data to be analyzed. Then, descriptive, bivariate and multivariate analyses were performed step by step by the researchers.

Descriptive statistics were used to calculate the mean and standard deviation for continuous variables and frequency for categorical variables. Bivariate analysis using cross-tabulation and bivariate logistic regression was done to determine distribution of the study subjects by independent variable of interest and to see crude associations between the independent variables and the dependent variables respectively, whereas multivariate analysis was used to evaluate independent effects of selected variables controlling the effects of others. Some variables, like parental educational status and occupation and religion, were re-categorized as there were no significant correlations established under the original categories used in the questionnaire.

### Ethical considerations

The protocol of this study was reviewed and approved by the Institutional Review Board of the College of Health Sciences at Addis Ababa University. After the endorsement by the review board, Hadiya zonal and woreda education officers and respective school principals were informed about the purpose of the study. In addition, informed consent was obtained from each study subject. Moreover, confidentiality and anonymity of the involved individual girls was maintained throughout the study.

## Results

### Socio-demographic characteristics of the study participants

Responses were obtained from 780 high school girls making the response rate 97.87%. The age of respondents range from 13–25 with a mean age of 16.2 (± 1.35 Standard Deviation). The majority of the respondents (64.9%), were from rural areas, 444 (56.9%) were grade 10 students, and 90% were Hadiya by ethnicity. Regarding the religion of the girls, 626 (80.3%) identified themselves as Protestant, 72 (9.2%) were Ethiopian Orthodox and 40 (5.1%) were Muslim (Table 1).

**Table 1:** Socio-demographic characteristics of high school girls in Hadiya zone, Ethiopia, 2011

**Variables**	**Number**	**Percent%**
**Age**		
Below 15 years	48	6.2
15–19 years	708	90.8
20–25 years	24	3.0
**Residence**		
Urban	274	35.1
Rural	506	64.9
**Grade level**		
Grade 9	336	43.1
Grade 10	444	56.9
**Religion**		
protestant	626	80.3
Orthodox	72	9.2
Muslim	40	5.1
Catholic	34	4.4
Others	5	0.6
No religion	3	0.4
**Ethnicity**		
Hadiya	694	89.0
Gurage	31	4.0
Kambata	28	3.6
Amhara	18	2.3
Silte	7	0.9
Others	2	0.3

(n = 780).

### Parental background information

Nearly half of the respondents’ fathers (49.8%) and more than half of the mothers (61.9%) had never attended school. Of the fathers, 180 (24.1%) and 182 (23.3%) of the mothers had attended elementary schools. Whereas the remaining 197 (25.3%) of the fathers and 103 (13.2%) of the mothers completed high school-level courses and above. The majority of the fathers (71.4%) were farmers whereas the majority of the mothers (70.4) were housewives (Table 2).

**Table 2:** Background information about the parents of high school girls in Hadiya zone, Ethiopia, 2011

**Variables**	**Numbers**	**Percent**
**Father’s educational status**		
Illiterate	189	24.2
Read and write only	200	25.6
1–8 grade	188	24.1
9–12 grade	123	15.8
Diploma and above	74	9.5
Do not know	6	0.8
**Mother’s educational status**		
Illiterate	274	35.1
Read and write only	209	26.8
1–8 grade	182	23.3
9–12 grade	78	10.0
Diploma and above	25	3.2
Do not know	12	1.5
**Father’s occupation**		
Farmer	557	71.4
Government employee	105	13.5
Private employee	93	11.9
No job	14	1.8
Others	11	1.4
**Mother’s occupation**		
Housewife	549	70.4
Farmer	92	11.8
Private employee	86	11.0
Government employee	51	6.5
Others	2	0.3

(n = 780).

### Prevalence, knowledge, attitudes and beliefs in the continuation of female genital cutting

#### Prevalence and knowledge on FGM

Half of the participants of the study responded that female genital cutting is currently being practiced in their area, 68 (8.7%) of them didn’t know whether it is practiced or not around their village. The majority (86.7%) stated that female genital cutting is harmful, 606 (77.7%) thought a girl has the right not to be circumcised.

Six-hundred-forty-one (82.2%) of the high school girls reported being circumcised (removal of the clitoris only) at a mean age of 11.0 (± 2.3 SD) years. More than half of the circumcisions (59.9%) were performed by traditional circumcisers (of whom 85.5% were male) while health professionals (Nurses and Health assistants) had also taken part in/performed (30%) of them. There were 429 (66.9%) group circumcisions and larger proportions (82.5%) were circumcised in their own homes (Table 3).

**Table 3:** Prevalence of FGC and knowledge on FGC among high school girls in Hadiya zone, Ethiopia, 2011

**Variables**	**Number**	**Percent**
**Circumcision status (n = 780)**		
Yes	641	82.2
No	139	17.8
**Operator/practitioner (n = 641)**		
Traditional birth attendant (TBA)	20	3.1
Traditional circumciser	384	59.9
Traditional circumciser who is also TBA	38	5.9
Health professional	186	29.0
Grandmother	1	0.2
Don’t know	12	1.9
**Sex of circumciser (n = 529)**		
Male	538	85.5
Female	91	14.5
**Condition of the circumcision (n = 641)**		
Group	429	67.0
Alone	190	29.6
Don’t know	22	3.4
**Presence of FGC in the village (n = 780)**		
Yes	391	50.1
No	321	41.2
Don’t know	68	8.7
**FGC is harmful practice (n = 780)**		
Yes	675	86.5
No	80	10.3
Don’t know	25	1.5
**A girl has the right not to be circumcised (n = 780)**		
Yes	606	77.7
No	162	20.8
Don’t know	12	1.5

#### Attitude and belief towards FGM

Among those who were circumcised, fewer than 10% supported their being circumcised. Of those who supported their circumcision, reasons for the support were mainly for hygienic purposes (35%), to avoid stigma of not being circumcised (31.6%) and out of respect for cultural norms (25%). Among those who opposed genital cutting their reason for their opposition was mostly because they consider FGC as a bad tradition. Six-hundred-eighty (87.2%) of the respondents had heard information from different sources concerning the harm of female genital cutting, the majority (53.8%) heard from health professionals. Only 41 (5.3%) of the high school girls who participated in this study believe the practice should be continued (Table 4).

**Table 4:** Attitudes and beliefs in the continuation of FGC among high school girls in Hadiya zone, Ethiopia, 2011

**Variables**	**Number**	**Percent**
**Support being circumcised (n = 641)**		
Support	60	9.4
Oppose	581	90.6
Reasons for supporting (n = 60)*		
To avoid shame and stigma	22	36.6
For hygiene	21	35.0
To respect culture	15	25.0
For religion	10	16.6
Preserve virginity	6	10.0
To avoid promiscuity	4	6.6
For better delivery of baby	3	5.0
Pressure from family	3	5.0
Better marriage prospect	2	3.0
Reasons for opposing (n = 581)*		
Bad tradition	388	66.8
Medical complications	307	52.8
Illegal	148	25.4
Painful process	108	18.7
Against women’s rights	78	13.4
**Heard message against FGC (n = 780)**		
Yes	680	87.2
No	100	12.8
**Believe in continuation (n = 780)**		
Yes	41	5.3
No	739	94.7

*More than one answer was possible.

### Factors associated with the prevalence of FGC

Logistic regression analysis was carried out to see the association of the socio-demographic and parental factors with the prevalence of the practice among the study participants.

In the bivariate analysis, residing in a rural area was positively and significantly associated with the prevalence of female genital cutting (COR = 2.6; 95% CI =1.79, 3.78). Female students at grade 10 were more likely to be circumcised compared to grade 9 students (COR = 1.59; 95% CI = 1.10, 2.29). Compared with Christian religions, the Muslim demonstrated a higher likelihood of being circumcised (COR = 4.21; 95% CI = 1.01, 17.00).

With regard to parental background information, having fathers with an educational status of below high school showed higher odds of being subjected to female genital cutting compared to those who had attended high school and above (COR = 3.02; 95% CI = 2.05, 4.45). The likelihood of circumcision was also higher among those who had mothers with an education level under high school (COR = 3.20; 95% CI = 2.03, 5.05). The odds of female genital cutting almost doubled among girls whose fathers were farmers compared to those whose fathers were currently employed in an organization (COR = 2.09; 95% CI = 1.42, 3.07). Having a housewife mother showed no association with the occurrence of female genital cutting (COR = 1.45; 95% CI = 0.92, 2.27), but there were higher odds of being subjected to FGC among those whose mothers were farmers (COR = 2.87; 95% CI = 1.30, 6.33) compared to those currently employed.

All variables associated (at significance level of 0.05) with the dependent variable under consideration in the bivariate analysis were also entered to multiple logistic regressions. Among those variables entered, four of them (residence, grade level, father’s education, and mother’s education) were found to have significant independent associations with the prevalence of female genital cutting. The remaining three (religion, father’s occupation and mother’s occupation) had no independent effects on the prevalence.

The odds of being cut was double among girls whose fathers had educational status under high school level when compared to those whose fathers had attended high school and above (AOR = 2.04; 95% CI: 1.25, 3.09). The odds of being subjected to the operation was 1.84 (95% CI: 1.01, 3.38) times higher among girls whose mothers had educational status of under high school level compared to those whose mothers had attended high schools and above (Table 5).

**Table 5:** Association of socio-demographic and parental factors with the prevalence of FGC among high school girls in Hadiya zone, Ethiopia, 2011

**Variables**	**Circumcision status**		**COR (95% CI)**	**AOR (95% CI)**
	**Yes**	**No**		
	**N (%)**	**N (%)**		
**Residence (n = 780)**				
Urban	199(72.6)	75(27.4)	1	1
Rural	442(87.4)	64(12.6)	2.6(1.79, 3.78)*	1.97(1.25, 3.09)*
**Grade level (n = 780)**				
Grade 9	263(78.3)	73(21.7)	1	1
Grade 10	378(85.1)	66(14.9)	1.59(1.10, 2.29)*	1.79(1.20, 2.66)*
**Religion (n = 772)**				
All Christian religions	599(81.8)	133(18.2)	1	1
Muslims	38(95.5)	2(5.0)	4.21(1.01,17.00)*	3.35(0.77, 14.5)
**Father’s educational status (n = 774)**				
Under high school	501(86.8)	76(13.2)	3.02(2.05, 4.45)*	2.04(1.25, 3.09)*
High school and above	135(68.5)	62(31.5)	1	1
**Mother’s educational status (n = 768)**				
Under high school	566(85.1)	99(14.9)	3.20(2.03, 5.05)*	1.84(1.10, 3.38)*
High school and above	66(64.1)	37(35.9)	1	1
**Father’s occupation (n = 766)**				
Farmer	474(85.1)	83(14.9)	2.09(1.42, 3.07)*	1.2(0.47, 1.44)
Currently employed	153(73.2)	56(26.8)	1	1
**Mother’s occupation (n = 780)**				
House wife	452(82.3)	97(17.7)	1.45(0.92,2.27)	1.06(0.51, 1.46)
Farmer	83(90.2)	9(9.8)	2.87(1.30, 6.33)*	1.49(0.63, 3.53)
Currently employed	106(73.3)	33(23.3)	1	1

*Significant at significance level of 0.05.

### Factors associated with beliefs in the continuation of female genital cutting among the high school girls

Female students who were from rural areas of the zone were more likely (COR = 2.31; 95% CI: 1.05, 5.09) to believe that female genital cutting should continue compared to those who were from the urban areas. Girls who responded that female genital cutting was being practiced in their village had higher odds of believing in its continuity (COR = 2.39; 95% CI: 1.14, 5.01) than those who responded that female genital cutting was not being practiced when this study was conducted. Odds of believing in the continuity of FGC were also higher among those who thought that female genital cutting is not harmful (COR = 5.75; 95% CI: 2.84, 11.65) and a girl doesn’t have the right to deny circumcision (COR = 5.13; 95% CI: 2.68, 9.82) compared to those who thought that female genital cutting is harmful and a girl has the right to deny, respectively.

Multivariate analysis of independent variables in relation to beliefs in the continuation of female genital cutting showed that presence of the practice in the area, thinking that FGC is harmful, thinking that a girl has a right not to be cut and the status of circumcision had an impact on belief in the continuation of the practice.

The odds of believing in the continuation of FGC was 2.33(95% CI: 1.01, 5.33) times higher among those who responded that FGC was practiced in their areas. Girls who thought that FGC is not harmful were 4.03(95% CI: 1.89, 8.59) times more likely to believe in the continuation of female genital cutting compared to those who thought that female genital cutting is harmful. Belief in the continuation of female genital cutting was high among high school girls who thought that a girl has no right to deny (AOR = 3.73; 95% CI: 1.81, 7.69). The odds of believing in the continuation of FGC was also 8.22 (95% CI: 1.10, 61.28) higher among circumcised girls compared to uncircumcised ones, but the confidence interval was very wide since the frequency of those who were not cut but believe in the continuation was only one (Table 6).

**Table 6:** Factors associated with beliefs in the continuation of FGC among high school girls in Hadiya zone, Ethiopia, 2011

**Variables**	**Belief in continuation**		**COR (95% CI)**	**AOR (95% CI)**
	**Yes**	**No**		
	**N (%)**	**N (%)**		
**Residence (n = 780)**				
Urban	8(2.9)	266(97.1)	1	1
Rural	33(6.5)	473(93.5)	2.32(1.05, 5.09)*	1.78(0.37, 1.65)
**Grade level (n = 780)**				
Grade 9	17(5.1)	319(94.9)	1	1
Grade 10	24(5.4)	420(94.6)	1.07(0.56, 2.03)	1.01(0.47, 2.03)
**Father’s educational status (n = 774)**				
Under high school	35(6.1)	542(93.9)	2.05(0.85, 4.96)	1.25(0.47, 2.03)
High school and above	6(3.0)	191(97.0)	1	1
**Mother’s educational status (n = 768)**				
Under high school	36(5.4)	629(94.6)	1.42(0.43, 2.92)	1.12(0.14, 2.13)
High school and above	5(4.9)	98(95.1)	1	1
**FGC is practiced in the area (n = 712)**				
Yes	28(7.2)	363(92.8)	2.39(1.14, 5.01)*	2.33(1.01, 5.33)*
No	10(3.1)	311(96.9)	1	1
**FGC is harmful (n = 755)**				
Yes	24(3.6)	615(96.4)	1	1
No	14(17.5)	66(82.5)	5.75(2.84, 11.65)*	4.03(1.89, 8.599)*
**A girl has right not to be cut(n = 768)**				
Yes	18(3.0)	588(97.0)	1	1
No	22(13.6)	140(86.4)	5.13(2.68, 9.82)*	3.73(1.81, 7.69)*
**FGC status (n = 780)**				
Yes	40(6.2)	601(93.8)	9.18(1.25, 67.38)**	8.22(1.10, 61.28)**
No	1(0.7)	138(99.3)	1	1

*Significant at significance level of 0.05.

**Significant at significance level of 0.05 but with less power.

## Discussion

In this study we found a very high prevalence of FGM in the area and circumcision of girls occurs at early adolescence with a mean age of 11 years. Girls support circumcision to avoid stigma associated with being uncircumcised. Apart from traditional circumcisers, health professionals were involved in female circumcision. Group circumcision was a common practice in the area.

The Ethiopian Penal Code states that female circumcision is illegal and punishable by law [15], However, eight out of ten girls reported having been circumcised, suggesting the practice is still in place. Combined with cultural taboo and stigma associated with being uncircumcised it seems less likely for the practice to vanish in the area in the near future. This figure is very high compared to both national and some other Sub-Saharan African countries. For example, the 2005 EDSH report indicates the total prevalence in Ethiopia and SNNPR was 74.3% and 71.0%, respectively [13], whereas the prevalence among the age group of 15–19 years in the whole country was 62.1% [13]. The rate reflected in this study was also extremely high compared to other East African nations. A study from Tanzania showed FGM prevalence among high school leavers was 10% and that of Kenya at 15% among age group of 15–19 years [4,16]. FGM prevalence within the same age cohort in other African countries was even higher for example 97% in Somalia [4].

A review of existing literature shows that the age of female circumcision in Ethiopia can vary greatly according to ethnicity and region. In the Amhara ethnic group/region, for example, circumcision might be initiated as early as eight days to one month after birth while Adere and Oromo conduct FGM among females aged from 4 years to puberty [17]. Other studies conducted in Hadiya Zone indicate the age of circumcision for girls can extend as high as 17 years [18]. In our study, however, circumcision of girls occurred at early adolescence with a mean age of 11 years.

Regarding pervasiveness of FGM in the Hadiya Zone, a 1996 baseline study showed the prevalence of female genital cutting among the Hadiya ethnic group as 74.8%. A follow-up national survey conducted 10 years later in 2006 showed a reduced prevalence of 70.9% [14]. Both studies reported lower numbers than the results found in this study. The difference in the magnitudes may be due to the age group of the samples included and/or the population sampled in each study. As most Hadiya girls are circumcised during early adolescence, this may have affected the estimates from the 1996 and the 2006 studies as the reports included younger, pre-adolescent children in their samples.

Similar to other studies [19-21], the main reasons given by the girls for the continuation of the practice were cultural considerations. This indicates the importance of culture, which rejects uncircumcised girls; it also supports other studies indicating that tradition and social importance were the main factors that subject a girl to circumcision [22,23]. The fact that a third of those who support their circumcised status did so for hygiene reasons suggests a continued belief in an effectiveness of FGC and therefore also a reduced likelihood of the termination of the practice. This was consistent with other studies [11,24]. Moreover, the involvement of health professionals in circumcising the girls may also contribute to the continuation as the community may think these silent referents were doing it for the enhancement of health.

The findings of the study have indicated that living in a rural area was associated with the high prevalence of FGC. This was consistent with Ethiopian DHS 2005 [13]. Studies from other countries have also shown that FGC prevalence is lower in urban communities [7,25]. Grade level of the girls also had an association with the prevalence of female genital cutting in this study with the odds of being circumcised greater among grade 10 female students compared to grade nine students. This may support a slight decrease in the prevalence over time. However, there is similar age aggregate in batch grade 9 and 10; thus, this study could not differentiate the variation in a significant way. According to a study done in Guinea, the effect of age on female genital cutting was insignificant [22].

Religion had no association with the occurrence of female genital cutting at multivariate level. Other studies also indicate that female genital cutting is not a result of religion and neither the Bible nor the Koran mention FGM as a religious requirement and most faiths, including Islam, forbid physical violation of the human body [12,19]. However, in other studies conducted in Sudan and in Ethiopia, followers of Muslim religion were more likely to be circumcised than other religions [12,23,26].

This study also indicated that increased education levels of both parents may contribute to the decrease of female genital cutting. Daughters from parents with an educational status below high school level were almost two times more likely to be circumcised compared with those with parental educational levels of high school and above, indicating the importance of parental education. In another study, it was also indicated that females with secondary education were four times more likely to oppose FGM [27] consistent with other studies [12,13,28,29].

With regard to belief in the continuation of FGC, only 5.3% of the study’s respondents believe in the continuation of the practice. This implies that if any intervention is taken to the community against the continuation of the practice, changes in behaviour and belief may be achieved.

The location of the participant’s residence also had significant association with the belief in the continuation of female genital cutting in the bivariate analysis, but it disappeared in the multivariate analysis. This was similar with the results of a study conducted in Guinea, where residence had no association with the belief in the continuation of FGC [30], but it was not consistent with a study performed in eastern Ethiopia [31].

The present study also found that being from the villages where female genital cutting is practiced influenced the belief in the continuation of the practice among high school girls. Those who responded that FGC was practiced in their area were 2.33 times more likely to support the continuation compared to those who responded that the practice had stopped or they didn’t know. So, those from villages where FGC is practiced had self-commitment and cultural pressure to believe in its continuation.

The effect of thinking FGC is harmful or not was highly significant in this study. Those who thought female genital cutting is not harmful were more likely to favour the continuation of the practice when compared with those who thought that FGC is harmful to the girl who undergoes the process. Similarly, another study also indicated that as the number of perceived advantages of female genital cutting increased, the tendency to support the discontinuation of FGC declined [30]. Thinking that a girl has the right to say ‘no’ to circumcision was also independently associated with the belief in the continuation. Those who thought a girl has no right to deny her own circumcision were more likely to believe in the continuation of FGC. This indicates that it is important to create awareness among students on the issues of human rights, including international conventions that condemn harmful practices and the Ethiopian penal code which outlaws female genital cutting [12,32,33].

Circumcision status was also found to be associated with the belief in the continuation of the practice in the multivariate analysis (AOR = 8.22; 95% CI: 1.10, 61.8). However, the effect size is not precise because of a small number of girls who were not circumcised and favoured the continuation. Similar results were obtained in other studies [17,30,32].

This study assessed the prevalence and belief in the continuation of Female Genital Cutting among high school girls. However it has certain limitation.

Since it was institution based study, this study could not link the prevalence with specific geographic area of the zone and self-reported information may be subjected to reporting errors, missed values and bias. Despite the above limitations, our findings are important to help to design appropriate interventions to halt the practice among the new generation of females in the area.

## Conclusion

From this study, we can conclude that the prevalence of FGM/FGC is still high in Ethiopia’s Hadiya Zone. Culture, stigma and hygiene were the most common reasons for the continuation of the practice. In addition, the fact that girls themselves demand to be circumcised to avoid shame and stigma is another reason for the continuation of the practice which should be handled carefully. As most of the girls were against the continuation of the practice regardless of their circumcision status, we can conclude that the practice will vanish if interventions are directed towards the alleviation of stigma at the community level. The persistence of the practice while the act is criminal is an issue that needs further study.

Local organizations in collaboration with religious institutions and community leaders should work together to engage in a process of change within the entire community by arranging awareness creation programmes on the harmfulness of the practice especially in the rural areas of the zone. Use of wider media coverage of positive role models, for example sports stars, fashion models and famous singers who have not been circumcised to become ambassadors for non-circumcised females could also break the shame and stigma. Moreover, there should be enforcement and implementation of the law to stop the health care workers from their collaboration with FGM.

## Competing interests

The authors declare that they have no competing interests.

## Authors’ contributions

MT and MM participated in study protocol development, data collection, analysis and write-up. Both authors read and approved the final manuscript.

## Pre-publication history

The pre-publication history for this paper can be accessed here:

http://www.biomedcentral.com/1471-2458/13/1120/prepub
